# Association between circadian rhythm sleep disorders and dry eye disease: a retrospective cohort study

**DOI:** 10.1038/s41598-026-67958-0

**Published:** 2026-09-09

**Authors:** Taher K. Eleiwa, J. Anthony Chacko, Reem H. ElSheikh, Qais A. Dihan, Mohammad Ayoubi, Ahmad Alzein, Hajirah N. Saeed, Subhi J. Al’Aref, Abdelrahman M. Elhusseiny

**Affiliations:** 1 Department of Ophthalmology, Benha University, Benha, Egypt; 2 Department of Ophthalmology, Jones Eye Institute, University of Arkansas for Medical Sciences, Little Rock, AR USA; 3 University of Miami Miller School of Medicine, Miami, FL USA; 4 Department of Ophthalmology, Illinois Eye and Ear Infirmary, University of Illinois at Chicago, Chicago, IL USA; 5 Department of Medicine, Division of Cardiology, University of Arkansas for Medical Sciences, Little Rock, AR USA; 6 Department of Ophthalmology, Boston Children’s Hospital, Harvard Medical School, Boston, MA USA

**Keywords:** Circadian Rhythm Sleep Disorder, Dry Eye Disease, Ocular Surface, Sleep Disturbance, Propensity Score Matching, Electronic Health Records, Diseases, Medical research, Risk factors

## Abstract

To evaluate the association between Circadian Rhythm Sleep Disorders (CRSD) and the risk of developing dry eye disease (DED). Retrospective cohort study using real-world clinical data. A total of 48,356 patients aged ≥ 18 years with a diagnosis of CRSD and 24,185 controls who underwent polysomnography and were not diagnosed with CRSD were identified. Electronic health records from the TriNetX U.S. Collaborative Network were accessed in March 2025. CRSD was identified using International Classification of Diseases-10 codes (G47.2), and patients with prior DED diagnoses were excluded. Propensity score matching (PSM) was conducted to control demographic, clinical, and pharmacologic confounders. The primary outcome was the risk of DED at 1-, 3-, and 5-year post-index date. Adjusted hazard ratios (aHR) and Kaplan-Meier survival analyses were used to evaluate the associations. Following 1:1 PSM, 19,877 patients were included in each cohort. Dry eye disease alone was significantly more frequent in the CRSD group at 1 year (0.58% vs. 0.22%; adjusted hazard ratio [aHR], 2.31; 95% CI, 1.85–2.87; *p* < 0.001), 3 years (1.25% vs. 0.61%; aHR, 2.12; 95% CI, 1.82–2.47; *p* < 0.001), and 5 years (1.58% vs. 0.89%; aHR, 2.04; 95% CI, 1.78–2.33; *p* < 0.001). The cumulative incidence of DED or initiation of DED medication was also elevated at 1 year (1.61% vs. 1.15%; aHR, 1.36; 95% CI, 1.22–1.53, *p* < 0.001), 3 years (3.15% vs. 2.65%; aHR 1.19; 95% CI, 1.1–1.29; *p* = 0.004), and 5 years (4.11% vs. 3.43%; aHR, 1.16; 95% CI, 1.08–1.25, *p* < 0.001). CRSD was associated with an increased risk of DED across all time points. These findings suggest a potential link between circadian disruption and ocular surface health, but should be interpreted with caution given the observational design.

**Supplementary Information:**

The online version contains supplementary material available at https://doi.org/10.1038/s41598-026-67958-0.

## Introduction

Circadian Rhythm Sleep Disorders (CRSD) are a group of chronic conditions characterized by a persistent misalignment between an individual’s endogenous circadian system and the external light-dark cycle, resulting in abnormal sleep timing and impaired daytime alertness^1^. CRSD are currently classified based on the International Classification of Sleep Disorders (ICSD-3), which distinguishes intrinsic disorders (e.g., delayed sleep–wake phase disorder, non-24-hour sleep–wake disorder) from extrinsic forms such as shift work and jet lag. CRSD includes specific subtypes such as delayed sleep-wake phase disorder, advanced sleep-wake phase disorder, irregular sleep-wake rhythm disorder, and non-24-hour sleep-wake disorder, each defined by a distinct disruption in the timing of the sleep-wake cycle rather than sleep architecture or quantity^2^.

Mounting evidence suggests that circadian dysregulation may have systemic effects beyond sleep, influencing metabolic, inflammatory, and neuroendocrine pathways that intersect with ocular surface homeostasis^3^. Notably, dry eye disease (DED), a chronic, multifactorial disorder of the ocular surface with global prevalence estimates ranging from 5% to over 50%, has been linked to disrupted sleep and circadian rhythm via mechanisms such as tear film instability, altered epithelial repair, and neuroimmune dysfunction^4–9^. Emerging literature supports a multifaceted connection between circadian regulation, ocular surface health, and sleep quality^3,10,11^.

Epidemiological studies have consistently shown that individuals with poor sleep characteristics, such as abnormal sleep duration or latency, are more likely to report dry eye symptoms, even after adjusting for systemic and psychiatric comorbidities^10–14^. Moreover, laboratory studies have demonstrated that sleep deprivation disrupts epithelial homeostasis and increases inflammatory mediators on the ocular surface^15^. In parallel, there is increasing recognition that DED may have a psychoneuroimmunological dimension, where factors such as mood disorders, fatigue, and circadian rhythm disturbances interact to exacerbate ocular discomfort^16,17^.

Despite these insights, there remains a lack of research specifically exploring how formally diagnosed CRSD impacts the risk of developing DED. Importantly, CRSDs are mechanistically and diagnostically distinct from other sleep disorders, such as obstructive sleep apnea, primary insomnia, or hypersomnia, which primarily involve sleep fragmentation, airway obstruction, or excessive total sleep, respectively, rather than circadian phase dysregulation^1,12^. This distinction is critical, as prior studies on DED have largely focused on general sleep impairment or sleep apnea without isolating the contribution of circadian misalignment, a gap this study aims to address^10,12,13,21–24^.

The present study aims to investigate the association between CRSD and the risk of DED using a large, multi-institutional, real-world dataset. Understanding this link could yield important insights into circadian-ocular interactions and inform targeted strategies for the prevention and management of DED in at-risk populations.

## Materials and methods

We conducted a retrospective cohort study using real-world clinical data from the TriNetX platform, which aggregates deidentified electronic health records from a wide range of healthcare organizations (HCOs). The database encompasses clinical, demographic, diagnostic, and procedural information for over 250 million patients globally. For this analysis, data was sourced from 69 participating United States-based HCOs within the United States Collaborative Network as of March 2025. TriNetX employs a federated real-world data platform that harmonizes diagnostic codes to common standards, including the International Classification of Diseases (ICD)-10. As part of this process, ICD-9 codes are internally converted to their ICD-10-CM equivalents to ensure consistency and comparability of clinical data across HCOs.

As the dataset was fully anonymized and compliant with the Health Insurance Portability and Accountability Act (HIPAA), the study qualified for exemption from Institutional Review Board (IRB) oversight at the University of Arkansas for Medical Sciences (UAMS). All procedures adhered to the ethical principles outlined in the Declaration of Helsinki (2013 version).

### Inclusion/exclusion criteria and study groups

The study population consisted of adults aged 18 years and older with a documented diagnosis of CRSD. CRSD was identified using ICD-10 code G47.2 and its subcodes, including delayed sleep-wake phase disorder, advanced sleep-wake phase disorder, irregular sleep-wake rhythm disorder, non-24-hour sleep-wake disorder, as well as shift work type and jet lag type circadian rhythm disorders. To enhance specificity, CRSD diagnosis required at least one documented ICD-10 G47.2 code in the absence of predefined exclusionary sleep disorders. Patients with a diagnosis of insomnia (ICD-10 G47.0); obstructive sleep apnea, adult or pediatric (ICD-10 G47.33); sleep deprivation (ICD-10 Z72.820); or morbid (severe) obesity with alveolar hypoventilation (ICD-10 E66.2) were excluded. A sensitivity analysis requiring 2 incidences of CRSD documentation was also included.

The control cohort included individuals aged 18 years or older who underwent polysomnography, identified by Current Procedural Terminology (CPT) codes 95808, 95810, or 95811, and who had no documented diagnosis of CRSDs (ICD-10 G47.2), sleep disorders (ICD-10 G47), sleep deprivation (ICD-10 Z72.820), or morbid (severe) obesity with alveolar hypoventilation (ICD-10 E66.2). The use of a polysomnography-based control group was intended to improve comparability in healthcare utilization and diagnostic intensity, thereby reducing potential detection bias inherent in electronic health record (EHR)-based studies.

A second general population control cohort included individuals aged 18 years or older who had a healthcare encounter (ICD-10 Z00.0) with no documented diagnosis of CRSDs (ICD-10 G47.2), sleep disorders (ICD-10 G47), sleep deprivation (ICD-10 Z72.820), or morbid (severe) obesity with alveolar hypoventilation (ICD-10 E66.2). Other non-circadian sleep disorders were not systematically excluded unless captured by these criteria.

The index date for CRSD patients was defined as the first occurrence of a qualifying CRSD diagnosis between January 1, 2010, and January 1, 2024. For control patients, the index date was the date of polysomnography, provided that all inclusion criteria were met. In both groups, we excluded patients who had a diagnosis of DED or were using DED topical therapy before the index event. Patients who entered the cohort closer to 2023 and lacked sufficient follow-up to reach the three- or five-year time points were excluded from the three-year outcome analysis but remained eligible for inclusion in the one-year analysis.

### Propensity score matching

To emulate randomized trial conditions and isolate the association between CRSD and the occurrence of DED, we performed 1:1 propensity score matching (PSM) using a nearest-neighbor matching algorithm without replacement. Propensity scores were calculated based on a comprehensive set of covariates, including demographic characteristics, lifestyle factors, clinical comorbidities, and medication use, each selected based on established associations with either CRSD, DED, or both. Baseline covariates and medication exposures were defined based on data available prior to or at the index date.

Demographic variables included age at index date, sex, race (White, Black or African American, Asian), and ethnicity (Hispanic or Latino, Non-Hispanic). Lifestyle factors such as nicotine dependence (ICD-10 F17) and overweight/obesity (ICD-10 E66) were included due to their potential impact on circadian regulation and ocular surface health.

Comorbid conditions accounted for in the matching process included psychiatric disorders (depression [ICD-10 F32] or anxiety [ICD-10 F41]), endocrine/metabolic diseases (diabetes mellitus [ICD-10 E08-E013], hypertension [ICD-10 I10], disorders of lipoprotein metabolism and other lipidemias [ICD-10 E78]), disorders of thyroid gland [ICD-10 E00-E07]), autoimmune diseases (Sjögren’s syndrome [ICD-10 M35.0], rheumatoid arthritis [ICD-10 M05, M06], vitiligo [ICD-10 L80], systemic lupus erythematosus [ICD-10 M32]), chronic lower respiratory diseases (ICD-10 J40-J4A), hypersensitivity disorders (Stevens-Johnson syndrome and toxic epidermal necrolysis [ICD-10 L51.1, L.51.2, L51.3]), and neurologic disorders such as Parkinson’s disease (ICD-10 G20).

Ocular conditions included in the matching process were lagophthalmos (ICD-10 H02.2), exophthalmic conditions (ICD-10 H05.2), disorders of the eyelid, lacrimal, and orbit (ICD-10 H00-H05), glaucoma (ICD-10 H40-H42), corneal disorders (corneal ulcer [ICD-10 H16.0], corneal neovascularization [ICD-10 H16.4], neurotrophic keratoconjunctivitis [ICD-10 H16.23], and corneal scars or opacities [ICD-10 H17]), anterior and posterior segment inflammation (iridocyclitis [ICD-10 H20], chorioretinal inflammation [ICD-10 H30]), pterygium of the eye (ICD-10 H11.0), and keratopathy following cataract surgery (ICD-10 H59.01). We also controlled for ocular surgical interventions, including cataract extraction (CPT 66982 and 66984), photorefractive keratectomy (CPT S0810), laser-assisted in situ keratomileusis (LASIK) (CPT S0800), chemodenervation of facial muscles (e.g., for blepharospasm, hemifacial spasm) (CPT 64612), intravitreal injections (CPT 67028), strabismus surgeries (CPT 67311–67312, CPT 67314–67318), retinal detachment surgeries (CPT 67036, 67039–67043, CPT 67108), and excision or transposition of pterygium (CPT 65420 and 65426). Including ocular conditions and ocular procedures was a deliberate strategy to control for baseline ocular disease burden and diagnostic intensity, with the understanding that this approach yields more conservative estimates.

Medication-related covariates included systemic drugs known to affect tear production or circadian rhythm. These included selective serotonin reuptake inhibitors (SSRIs) (ATC N06AB), tricyclic antidepressants (VA CN601), monoamine reuptake inhibitors (ATC N06AA), antihistamines (VA AH000), beta-blockers (ATC C07), diuretics (ATC C03), anticholinergics (e.g., tolterodine (RxNorm 119565), oxybutynin (RxNorm 32675)), HMG CoA reductase inhibitors (ATC C10AA) and isotretinoin (RxNorm 6064).

Covariate balance before and after matching was assessed using standardized mean differences (SMDs). To adjust for potential overadjustment in the matching process, we included a sensitivity analysis excluding healthcare utilization variables (e.g., ophthalmology visits) from the matching model.

### Study outcomes

Two outcome definitions were evaluated: (1) DED diagnosis alone, and (2) a composite of DED diagnosis or initiation of topical therapy. DED diagnoses were ascertained via ICD-10 codes for keratoconjunctivitis sicca (H16.22) and dry eye disease (H04.12). Medications considered indicative of DED treatment included ophthalmic cyclosporine (Restasis or Cequa) (RxNorm 3088), lifitegrast (RxNorm 1801820), varenicline (RxNorm 591622), carboxymethylcellulose (RxNorm 2062), glycerin (RxNorm 4910), polyvinyl alcohol (RxNorm 8570), polyethylene glycol (RxNorm 8514), or dextran 70 (RxNorm 3274). In the composite outcome, we included the percentage of outcomes detected via DED and medication initiation in a supplemental material. Because DED diagnoses and medication initiation were jointly used to define the composite outcome, and patients could meet one or both criteria, composite percentages are not mutually exclusive and do not sum to the overall composite incidence.

### Statistical analysis

To assess the effectiveness of the PSM, we compared baseline characteristics between cohorts using SMDs, with values below 0.1 indicating acceptable covariate balance. After matching, we used descriptive statistics to summarize baseline characteristics, with continuous variables presented as means ± standard deviations (SD) and categorical variables as frequencies and percentages.

Adjusted hazard ratios (aHRs) and corresponding 95% confidence intervals (CIs) were calculated to estimate the association between CRSD and the development of DED, accounting for matched covariates. The proportional hazards assumption was assessed using the generalized Schoenfeld method and was not violated. Robustness of the estimates to the sample-size reduction inherent in propensity-score matching was evaluated through sensitivity analyses, described below.

Within each time-specific cohort, Kaplan–Meier survival analyses and Cox proportional hazards models were applied, with appropriate censoring of patients who did not experience the outcome during the available follow-up period. All statistical analyses were conducted using the TriNetX platform, with significance defined at a two-sided p-value < 0.05.

## Results

### Baseline characteristics

Prior to PSM, the study included 48,356 patients with a diagnosis of CRSD and 24,185 patients in the unmatched polysomnography control cohort. The mean age at the index event was comparable between groups: 39.02 ± 20.5 years for CRSD patients and 41.72 ± 20.8 years for controls. Demographically, the CRSD group had a slightly higher proportion of females (54.3%) compared to the control group (51.7%). The distribution of race and ethnicity in the CRSD group was 69.4% White (57.5% in the polysomnography group), 14.2% Black or African American (18.5% in the PSG control), 3.7% Asian (1.6% in the polysomnography control), and 71.0% Not Hispanic or Latino (58.8% in the polysomnography control); Hispanic or Latino ethnicity was recorded in 6.5% of the CRSD group and 15.8% of controls. Comorbid conditions were more prevalent in the CRSD group, with 22.1% having hypertension (18.9%), 8.4% diabetes (7.4%), 27% anxiety disorders (9.5%), 20.5% depressive episodes (7.9%), and 8.5% with nicotine dependence (5.6%).

**Fig. 1:**
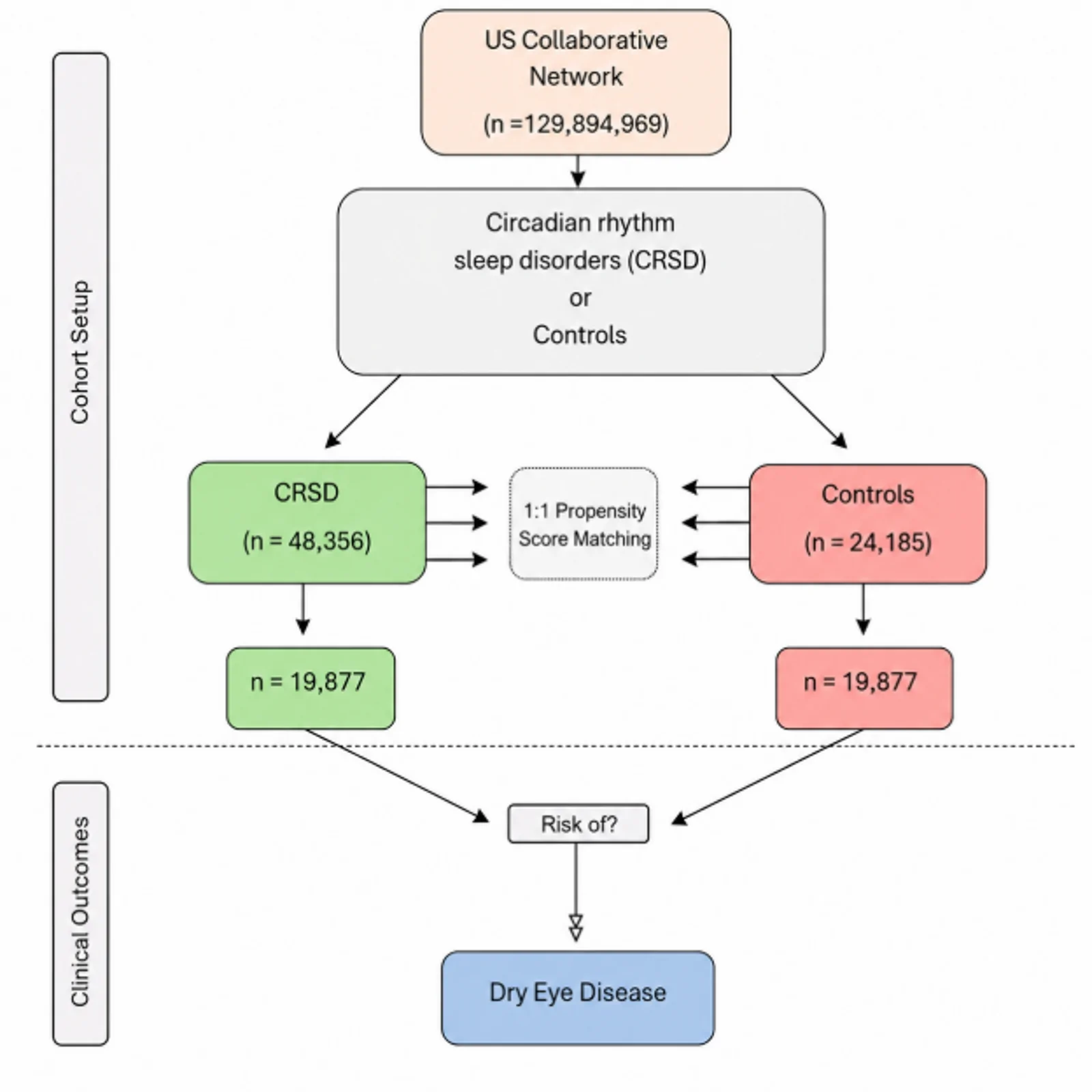
Flow chart illustrating the categorization of a study population.

**Fig. 2:**
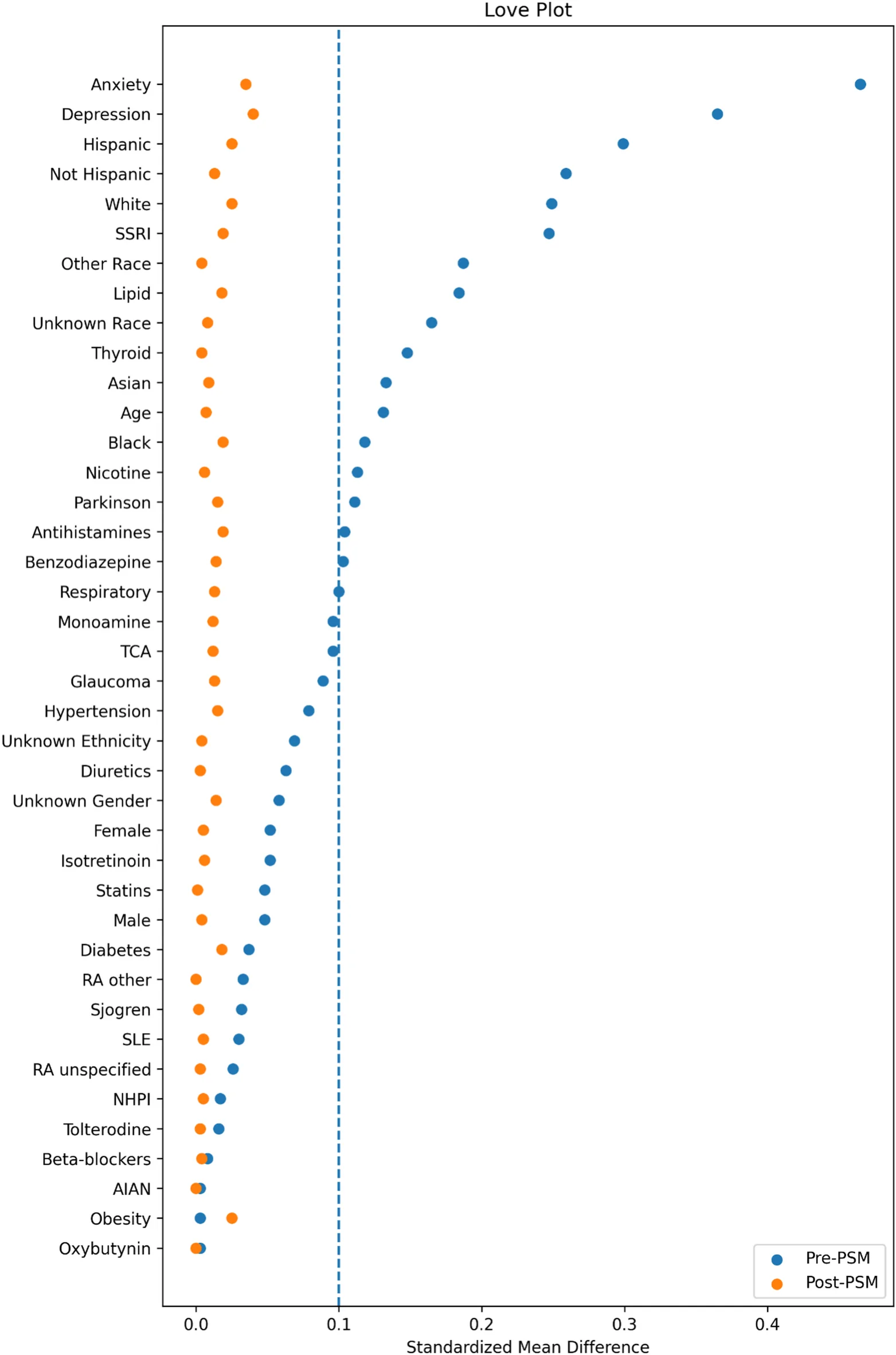
Love plot demonstrating standardized mean differences for all covariates before and after propensity score matching. All post-matching values were < 0.1, indicating adequate balance.

Following 1:1 PSM using nearest-neighbor matching, 19,877 patients were included in each group (Fig. 1**).** Matching resulted in balanced cohorts across baseline covariates (see Table 1**).** All covariates achieved standardized mean differences < 0.1 after matching, confirming adequate balance (Fig. 2). Due to the use of fixed-horizon analyses within the TriNetX platform, only patients with sufficient observable follow-up contributed to each time-point analysis. Exact numbers at risk for each horizon were not available from the aggregate TriNetX output; however, analyses were conducted within the matched cohorts of 19,877 patients per group, with patients lacking sufficient follow-up for a given horizon excluded from that specific time-point analysis.

The distribution of CRSD subtypes in the experimental cohort after matching was 29.8% shift work, 18.5% delayed sleep phase, 4.9% jet lag, 3.7% irregular sleep wake, 1.4% advanced sleep phase, and 40.3% unspecified (see Table 2). Because unspecified and shift-work types together accounted for the majority of cases, these findings should be interpreted as an association with clinically coded CRSD as a composite, EHR-based exposure rather than a subtype-specific effect of circadian misalignment; subtype-level effects could not be isolated.

## Association between circadian rhythm sleep disorder and dry eye disease

### Circadian rhythm sleep disorders vs. polysomnography control

The development of DED was significantly higher in the CRSD group across all follow-up intervals compared to the polysomnography control group. At 1 year, the frequency of a new DED diagnosis was 0.58% compared to 0.22% in the polysomnography group (*p* < 0.001); at 3 years, 1.25% vs. 0.61% (*p* < 0.001), and at 5 years, 1.58% vs. 0.89% (*p* < 0.001). The observation of DED or the initiation of DED medication was also elevated at 1 year (1.61% vs. 1.15%, *p* < 0.001), 3 years (3.15% vs. 2.65%, *p* = 0.004), and 5 years (4.11% vs. 3.43%, *p* < 0.001), with the proportion of composite outcomes ascertained by DED diagnosis versus topical therapy initiation is presented in Supplementary Table 1.

In a sensitivity analysis excluding healthcare-utilization variables from the matching model to assess potential over-adjustment, the direction and significance of the association were unchanged: a new DED diagnosis remained more frequent in the CRSD cohort at 1, 3, and 5 years (0.56% vs. 0.20%; 1.30% vs. 0.55%; 1.74% vs. 0.82%; all *p* < 0.001, respectively), as did the composite outcome (1.64% vs. 1.12%; 3.34% vs. 2.56%; 4.38% vs. 3.44%; all *p* < 0.001, respectively), consistent with the primary matched analysis.

Cox proportionality hazard showed an increased adjusted hazard in developing DED at 1 year (aHR: 2.31, 95% CI: 1.85–2.87, *p* < 0.001), 3 years (aHR: 2.12, 95% CI: 1.82–2.47, *p* < 0.001), and 5 years (aHR: 2.04, 95% CI: 1.78–2.33, *p* < 0.001). Additionally, there was an increased hazard in developing DED or initiating DED medication among the CRSD cohort compared to the polysomnography cohort at 1 year with an aHR 1.36 (95% CI: 1.22–1.53, *p* < 0.001), 3 years (aHR: 1.19, 95% CI: 1.1–1.29, *p* < 0.001) and 5 years (aHR: 1.16, 95% CI: 1.08–1.25, *p* < 0.001).

### Circadian rhythm sleep disorders vs. general population control

In comparison to the general population, the frequency of a new DED diagnosis remained elevated at 1 year (0.57% vs. 0.4%, *p* < 0.001), 3 years (1.31% vs. 0.95%, *p* < 0.001), and 5 years (1.78% vs. 1.32%, *p* < 0.001). The observation of DED or initiation of DED medication was similarly elevated at 1 year (1.68% vs. 1.15%, *p* < 0.001), 3 years (3.32% vs. 2.6%, *p* < 0.001), and 5 years (4.37% vs. 3.58%, *p* < 0.0001). A Cox proportionality hazards model showed elevated hazard of DED diagnosis compared to the general population at 1 year (aHR: 1.29, 95% CI: 1.18–1.41, *p* < 0.001), 3 years (aHR 1.23, 95% CI: 1.16–1.31, *p* < 0.001) and 5 years (aHR: 1.2, 95% CI: 1.13–1.27, *p* < 0.001). Hazards of developing DED or initiating medication was elevated in the CRSD cohort at 1 year (aHR: 1.5, 95% CI: 1.42–1.59, *p* < 0.001), 3 year (aHR: 1.29, 95% CI: 1.24–1.34, *p* < 0.001), and 5 years (aHR: 1.24, 95% CI: 1.19–1.28 *p* < 0.001).

### Sensitivity analysis

In the sensitivity analysis requiring 2 instances of CRSD diagnosis in the experimental cohort, the frequency of a new DED diagnosis remained elevated at 1 year (0.6% vs. 0.25%, *p* < 0.001), 3 years (1.25% vs. 0.77%, *p* < 0.001), and 5 years (1.54% vs. 1.13%, *p* = 0.006). A Cox proportionality hazards model showed an increased hazard in developing DED in the CRSD cohort at 1 year (aHR: 2.54, 95% CI: 1.96–3.27, *p* < 0.001), 3 years (aHR: 2.13, 95% CI: 1.77–2.55, *p* < 0.0001), and 5 years (aHR: 1.98, 95% CI: 1.69–2.37, *p* < 0.001). Additionally, the hazard of developing DED or initiating medication was higher in the CRSD cohort at 1 year (aHR: 1.19, 95% CI: 1.03–1.37, *p* = 0.01), 3 years (aHR: 1.15, 95% CI: 1.04–1.28, *p* = 0.007), and 5 years (aHR: 1.12, 95% CI: 1.01–1.22, *p* = 0.02). Sensitivity analyses using diagnosis-based definitions alone yielded consistent results across all time points, supporting the robustness of the primary findings.

## Discussion

In this retrospective cohort study, we observed a significant association between CRSD and an elevated hazard of developing DED. The aHRs ranged from 1.16 to 2.54 depending on the comparator and outcome definition, with higher estimates observed when compared to the polysomnography control cohort. The magnitude of the association also varied with the outcome definition. Effect estimates were consistently larger for the diagnosis-only outcome (aHR 2.04–2.31 versus the polysomnography cohort) than for the composite of DED diagnosis or topical-therapy initiation (aHR 1.16–1.36), while remaining statistically significant under both definitions. This indicates that the outcome definition itself influences the estimated strength of the association. The most plausible explanation is differential specificity: a coded DED diagnosis reflects a clinician-confirmed disease state, whereas the composite additionally captures initiation of topical agents, particularly over-the-counter lubricants, that are frequently used for transient or non-specific ocular surface symptoms unrelated to chronic DED. Because such therapy use is common in both exposed and control patients, its inclusion broadens the outcome to a less specific phenotype and biases the exposure–outcome association toward the null, yielding a smaller hazard ratio. Consistent with this, the medication-initiation component alone did not differ significantly between the CRSD and polysomnography cohort. The diagnosis-based estimate therefore likely provides a more specific measure of the CRSD–DED association, whereas the composite offers greater sensitivity at the cost of specificity.

The increased risk remained robust following PSM and the Cox proportional hazards model and in an additional sensitivity analysis, supporting an association between CRSD and increased risk of DED. Hazard ratios were somewhat higher in the first year than at five years. This pattern should be interpreted cautiously and may partly reflect detection bias, whereby increased healthcare engagement after a CRSD diagnosis leads to earlier identification of DED, and depletion of susceptible individuals earlier in follow-up; a larger short-term contribution of circadian disruption is also possible. These explanations are not mutually exclusive and cannot be distinguished in the present data.

Our findings expand upon a growing body of epidemiological research demonstrating a bidirectional relationship between sleep disturbances and DED symptoms. In a population-based study of 3,303 Singaporean Malay and Indian adults, Lim et al. reported that short sleep duration (odds ratio (OR) = 1.73), clinical insomnia (OR = 3.68), sleep apnea (Berlin Questionnaire OR = 1.55; STOP-Bang OR = 2.66), and excessive daytime sleepiness (OR = 1.77) were each independently associated with DED symptoms, even after adjusting for mood disorders, asthma, and medication use^21^. This was followed by a large-scale European cohort study by Magno et al., which analyzed data from over 71,000 individuals in the Lifelines cohort and found that patients with DED were significantly more likely to report poor sleep quality, with elevated Pittsburgh Sleep Quality Index (PSQI) scores and prolonged sleep latency, even after adjusting for more than 50 comorbidities^11^. Most recently, a 2024 meta-analysis by Gu et al., synthesizing data from 21 observational studies and 419,218 participants, confirmed that DED patients had over twice the risk of sleep disorders (relative risk (RR) = 2.20), with significantly increased risks for both insufficient sleep (RR = 3.76) and excessive sleep (RR = 5.53), along with worse subjective sleep quality (PSQI weighted mean difference (WMD) = 1.78) and greater daytime sleepiness (Epworth Sleepiness Scale (ESS) WMD = 3.02).^10^ These findings are further supported by additional population-based studies. Zheng et al., in a large retrospective cohort, demonstrated that patients with sleep disorders had a significantly increased risk of developing incident DED, reinforcing the temporal relationship between sleep dysfunction and ocular surface disease^26^. Similarly, Hanyuda et al., using data from the Japan Public Health Center-based Prospective Study (JPHC-NEXT), reported that shorter sleep duration and poor sleep quality were associated with a higher prevalence of DED symptoms^27^. Together, these studies further support the link between sleep disturbance and ocular surface dysfunction across diverse populations and study designs. However, despite a consistent and growing body of evidence linking various sleep disorders to DED, no prior study, to our knowledge, has specifically examined the association between CRSD and DED risk in a real-world cohort. Results from this distinction are consistent with the possibility that clinically coded CRSD may be associated with subsequent DED risk, although the present study cannot determine whether circadian misalignment itself directly contributes to ocular surface dysfunction.

Unlike glaucoma, where circadian misalignment affects intraocular pressure regulation and optic nerve perfusion, the mechanisms by which CRSD contributes to DED may be more rooted in peripheral circadian desynchrony, such as impaired clock gene expression in ocular surface tissues^5,18-20^. Recent studies have revealed the expression of Brain and Muscle ARNT-Like 1(BMAL1), Circadian Locomotor Output Cycles Kaput (CLOCK), and Period Circadian Regulator 1 (PER1), core components of the circadian clock, in conjunctival and meibomian gland cells, governing local rhythms in tear film dynamics and inflammation^5,8,25^. Chronic circadian disruption may attenuate this transcriptional machinery driving circadian oscillations, resulting in persistent dry eye symptoms despite normal ocular findings^3^. For example, BMAL1 overexpression increases mucin 4 levels in human corneal epithelial cells, while its deletion exacerbates epithelial damage and inflammation^8^. These experimental findings from other studies offer biologically plausible pathways that could underlie such an association, though they were not evaluated in our study.

Furthermore, our findings also reinforce the psychiatric and neurologic dimensions of DED. A “vicious spiral” has been proposed in which DED, sleep disorders, and mood disturbances reinforce one another, contributing to a self-perpetuating cycle of inflammation, neurosensory dysregulation, and circadian misalignment^6^. Our findings extend prior observations by demonstrating a significant association between clinically coded CRSD and DED in a large real-world cohort.

This emerging “sleep–tear axis” underscores the complexity of DED pathogenesis. These findings raise the possibility that sleep and circadian health may be relevant to DED risk assessment, but therapeutic implications require prospective and mechanistic validation. Our findings may also support consideration of sleep health in patients with chronic or refractory dry eye symptoms. The association between CRSD and DED raises important questions about the timing of symptom onset, the potential benefit of chronotherapy, and the role of multidisciplinary management. Although the observed effect sizes were modest, they were consistent across analyses and likely reflect the contribution of circadian dysregulation within the broader, multifactorial pathophysiology of DED. Accordingly, these findings should be interpreted as hypothesis-generating and require confirmation in prospective and mechanistic studies.

While our study leveraged a large, real-world, multi-institutional dataset and applied robust PSM to minimize confounding, several limitations should be acknowledged. First, as a retrospective observational analysis, the study design precludes definitive causal inference. Additionally, CRSD identification relied on ICD-10 coding without validation through repeated diagnoses or specialist confirmation, which may introduce misclassification bias, especially for subtypes of CRSD and milder or unrecorded cases of DED, and may obscure differences in dry eye risk across biologically distinct CRSD subtypes. Accordingly, our findings reflect the risk associated with a composite, EHR-coded CRSD phenotype and should not be interpreted as evidence of a subtype-specific effect of circadian misalignment. Because unspecified and shift-work types predominated, subtype-level mechanisms, which differ between intrinsic circadian disorders and extrinsic forms such as shift work and jet lag, could not be disentangled, and future work should examine whether specific subtypes confer differential DED risk. Given the heterogeneity of CRSDs and potential differences in underlying mechanisms, future studies should explore whether specific CRSD subtypes confer differential risk for DED. As with all EHR-based datasets, the TriNetX platform relies on accurate diagnoses and documentation through proper ICD codes. Although we used a comprehensive diagnostic and treatment-based case definition for DED, variations in documentation and coding practices across institutions could affect case capture. Additionally, the inclusion of topical therapies, particularly over-the-counter lubricants, as part of the DED definition may reduce specificity, as these agents can be used for transient or non-specific ocular surface symptoms. We also ran a sensitivity analysis enforcing multiple CRSD diagnoses, which yielded consistent results and supported the robustness of our study, suggesting that misclassification is unlikely to fully explain the observed association. Another limitation is that CRSD was treated as a time-fixed exposure based on the initial diagnosis, and changes in circadian rhythm status over time could not be captured. As circadian disorders may improve or fluctuate, this may introduce non-differential exposure misclassification, potentially biasing results toward the null. Future studies incorporating time-updated exposure definitions and circadian biomarkers are warranted to better characterize the dynamic relationship between CRSD and ocular surface disease. Additionally, the TriNetX platform does not provide access to patient-reported outcomes or structured symptom questionnaires, such as the Ocular Surface Disease Index (OSDI), nor does it include objective sleep metrics (e.g., actigraphy). This limits granularity in characterizing circadian disruption severity or DED symptom burden. In addition, despite extensive matching, residual confounding remains likely, as several relevant factors, including screen exposure, occupational shift work patterns, contact lens use, environmental conditions, medication intensity over time, psychiatric disease severity, and healthcare-seeking behavior, are not fully captured in EHR-based datasets. Furthermore, the control group consisted of patients undergoing polysomnography, which may represent a clinically selected population rather than a general healthy cohort. While this approach improves comparability in healthcare engagement, it may introduce a conservative bias, as these individuals may have underlying sleep-related symptoms that could independently increase DED risk, thereby attenuating observed associations. Importantly, sensitivity analyses using an alternative general population control cohort yielded consistent results, supporting the robustness of our findings. Additionally, the estimated HRs attenuated across follow-up. Although the proportional hazards assumption was not rejected by the generalized Schoenfeld test, this test does not by itself establish that the time-varying pattern is free of bias; the attenuation may reflect detection bias or differential outcome ascertainment over time rather than a true change in effect, and should be regarded as a limitation requiring confirmation in future work. Finally, while the study population was diverse and derived from multiple United .States-based healthcare organizations, the results may not generalize to populations outside the United States or to patients receiving care in non-integrated or community-based health systems. Future prospective studies incorporating multimodal sleep assessment, circadian biomarkers, and patient-reported symptomatology are needed to confirm these findings and better define the mechanisms linking CRSD to DED.

In conclusion, CRSD was associated with an increased risk of DED in this large retrospective cohort. These findings suggest a potential link between circadian disruption and ocular surface health but should be interpreted with caution given the observational design and potential for residual confounding.

**Table 1:** Baseline characteristics of the circadian rhythm sleep disorder (CRSD) cohort and matched polysomnography-based control cohort (PSG) before and after propensity score matching (PSM).

Characteristic Name	Pre-PSM			Post-PSM		
	CSRD Cohort	PSG Control	Standardized	CSRD	PSG Control	Standardized
	(*n*= 48,356)	(*n*=24,185)	Mean Difference	(*n*=19,877)	(*n*=19,877)	Mean Difference
Age at Index, mean (SD)	39.02 (20.5)	41.72 (20.77)	0.131	40.85 (21.2)	40.7 (20.3)	0.007
**Gender**						
Male, n (%)	22,084 (45.7%)	11,621 (48.1%)	0.048	9449 (47.5%)	9487 (47.7%)	0.004
Female, n (%)	26,247 (54.3%)	12,494 (51.7%)	0.052	10,406 (52.4%)	10,358 (52.1%)	0.005
Unknown Gender, n (%)	25 (0.1%)	70 (0.3%)	0.058	22 (0.1%)	32 (0.2%)	0.014
**Ethnicity**						
Hispanic or Latino, n (%)	3143 (6.5%)	3824 (15.8%)	0.299	2355 (11.8%)	2197 (11.1%)	0.025
Not Hispanic or Latino, n (%)	34,356 (71.0%)	14,216 (58.8%)	0.259	12,667 (63.7%)	12,790 (64.3%)	0.013
Unknown Ethnicity, n (%)	10,857 (22.5%)	6145 (25.4%)	0.069	4855 (24.4%)	4890 (24.6%)	0.004
**Race**						
White, n (%)	33,553 (69.4%)	13,905 (57.5%)	0.249	12,074 (60.7%)	12,317 (62.0%)	0.025
Black or African American, n (%)	6855 (14.2%)	4485 (18.5%)	0.118	3707 (18.7%)	3563 (17.9%)	0.019
Asian, n (%)	1769 (3.7%)	375 (1.6%)	0.133	399 (2.0%)	373 (1.9%)	0.009
Native Hawaiian or Other Pacific Islander, n (%)	194 (0.4%)	125 (0.5%)	0.017	113 (0.6%)	106 (0.5%)	0.005
American Indian or Alaska Native, n (%)	214 (0.4%)	112 (0.5%)	0.003	94 (0.5%)	94 (0.5%)	0
Unknown Race, n (%)	3950 (8.2%)	3204 (13.2%)	0.165	2282 (11.5%)	2233 (11.2%)	0.008
Other Race, n (%)	1821 (3.8%)	1979 (8.2%)	0.187	1208 (6.1%)	1191 (6.0%)	0.004
**Comorbid Conditions**						
Essential (primary) hypertension, n (%)	10,672 (22.1%)	4564 (18.9%)	0.079	4240 (21.3%)	4121 (20.7%)	0.015
Diabetes mellitus, n (%)	4041 (8.4%)	1781 (7.4%)	0.037	1660 (8.4%)	1565 (7.9%)	0.018
Disorders of thyroid gland, n (%)	5170 (10.7%)	1584 (6.6%)	0.148	1508 (7.6%)	1486 (7.5%)	0.004
Disorders of lipoprotein metabolism and other lipidemias, n (%)	10,621 (22.0%)	3593 (14.9%)	0.184	3526 (17.7%)	3388 (17.0%)	0.018
Chronic lower respiratory diseases, n (%)	7709 (15.9%)	3009 (12.4%)	0.1	2776 (14.0%)	2685 (13.5%)	0.013
Other rheumatoid arthritis, n (%)	456 (0.9%)	158 (0.7%)	0.033	146 (0.7%)	146 (0.7%)	0
Rheumatoid arthritis, unspecified,, n (%)	341 (0.7%)	121 (0.5%)	0.026	114 (0.6%)	109 (0.5%)	0.003
Parkinson’s disease, n (%)	521 (1.1%)	47 (0.2%)	0.111	63 (0.3%)	47 (0.2%)	0.015
Systemic lupus erythematosus (SLE), n (%)	210 (0.4%)	62 (0.3%)	0.03	63 (0.3%)	58 (0.3%)	0.005
Sjögren syndrome, n (%)	189 (0.4%)	52 (0.2%)	0.032	49 (0.2%)	51 (0.3%)	0.002
Overweight and obesity, n (%)	8203 (17.0%)	4132 (17.1%)	0.003	3744 (18.8%)	3553 (17.9%)	0.025
Other anxiety disorders, n (%)	13,049 (27.0%)	2293 (9.5%)	0.465	2499 (12.6%)	2272 (11.4%)	0.035
Depressive episode, n (%)	9904 (20.5%)	1919 (7.9%)	0.365	2113 (10.6%)	1876 (9.4%)	0.04
Nicotine dependence, n (%)	4100 (8.5%)	1351 (5.6%)	0.113	1275 (6.4%)	1244 (6.3%)	0.006
Glaucoma, n (%)	948 (2.0%)	218 (0.9%)	0.089	232 (1.2%)	206 (1.0%)	0.013
**Medications**						
Antihistamines, n (%)	13,630 (28.2%)	5721 (23.7%)	0.104	5165 (26.0%)	5004 (25.2%)	0.019
Benzodiazepine, n (%)	12,319 (25.5%)	5107 (21.1%)	0.103	4581 (23.0%)	4461 (22.4%)	0.014
B-blockers, n (%)	6883 (14.2%)	3509 (14.5%)	0.008	2877 (14.5%)	2906 (14.6%)	0.004
Diuretics, n (%)	5807 (12.0%)	3421 (14.1%)	0.063	2778 (14.0%)	2754 (13.9%)	0.003
HMG CoA reductase inhibitors, n (%)	5861 (12.1%)	3320 (13.7%)	0.048	2669 (13.4%)	2674 (13.5%)	0.001
Selective serotonin reuptake inhibitors, n (%)	9594 (19.8%)	2657 (11.0%)	0.247	2566 (12.9%)	2439 (12.3%)	0.019
Non-selective monoamine reuptake inhibitors, n (%)	2409 (5.0%)	750 (3.1%)	0.096	739 (3.7%)	695 (3.5%)	0.012
Tricyclic Antidepressants, n (%)	2409 (5.0%)	749 (3.1%)	0.096	739 (3.7%)	694 (3.5%)	0.012
Oxybutynin, n (%)	666 (1.4%)	324 (1.3%)	0.003	277 (1.4%)	276 (1.4%)	0
Tolterodine, n (%)	203 (0.4%)	78 (0.3%)	0.016	71 (0.4%)	68 (0.3%)	0.003
Isotretinoin, n (%)	148 (0.3%)	19 (0.1%)	0.052	23 (0.1%)	19 (0.1%)	0.006

**Table 2:** Distribution of CRSD subtypes within the CRSD (exposed) cohort. Subtypes were not used as matching covariates and are not compared with controls. Subtypes are not mutually exclusive; some patients had more than one subtype diagnosis. Therefore, subtype counts exceed the total cohort size and percentages sum to more than 100%.

Subtype	Pre-PSM (*n*=48,356)	Post-PSM (*n*=19,877)
Circadian rhythm sleep disorder, unspecified type	19,505 (40.3%)	8,126 (40.9%)
Circadian rhythm sleep disorder, shift work type	14,388 (29.8%)	5,815 (29.3%)
Circadian rhythm sleep disorder, delayed sleep phase type	8,945 (18.5%)	3,561 (17.9%)
Circadian rhythm sleep disorder, jet lag type	2,363 (4.9%)	979 (4.9%)
Circadian rhythm sleep disorder, irregular sleep wake type	1,799 (3.7%)	749 (3.8%)
Circadian rhythm sleep disorder, advanced sleep phase type	668 (1.4%)	351 (1.8%)
Circadian rhythm sleep disorder in conditions classified elsewhere	336 (0.7%)	163 (0.8%)
Circadian rhythm sleep disorder, free running type	233 (0.5%)	82 (0.4%)
Other circadian rhythm sleep disorder	585 (1.2%)	236 (1.2%)

## Supplementary Information

Below is the link to the electronic supplementary material.

Supplementary Material 1

## Data Availability

The datasets generated and/or analyzed during the current study are available in the TriNetX research network repository, https://trinetx.com. The data are not publicly available as they are from a licensed subscription-based platform containing proprietary electronic health records. Access is restricted to authorized users at participating institutions, and therefore, no accession numbers are available.

## Declarations

### Data statement

Data is available through the TriNetX online database.
