# Supplementary material for: Association between circadian rhythm sleep disorders and dry eye disease: a retrospective cohort study
Source: Sci Rep. 2026 Sep 9;16:28118. doi: 10.1038/s41598-026-67958-0 (PMC13554225; doi:10.1038/s41598-026-67958-0)
Supplement: Supplementary file 1 [file 41598_2026_67958_MOESM1_ESM.docx]

| Follow-up | CRSD | Control |  | CRSD | Control |  |
| --- | --- | --- | --- | --- | --- | --- |
|  | **DED diagnosis, n (%)** | **DED diagnosis, n (%)** | **P-value** | **Medication initiation, n (%)** | **Medication initiation, n (%)** | **P-value** |
| CRSD vs PSG (n=19,877) |  |  |  |  |  |  |
| 1 year | 116 (0.58%) | 44 (0.22%) | <0.001 | 220 (1.11%) | 183 (0.92%) | =0.078 |
| 3 years | 249 (1.25%) | 122 (0.61%) | <0.001 | 415 (2.08%) | 420 (2.11%) | =0.76 |
| 5 years | 314 (1.58%) | 177 (0.89%) | <0.001 | 564 (2.8%) | 537 (2.7%) | =0.51 |
| CRSD vs General Population (n=48,356) |  |  |  |  |  |  |
| 1 year | 277 (0.57%) | 195 (0.4%) | <0.001 | 579 (1.2%) | 391 (0.81%) | <0.001 |
| 3 years | 632 (1.31%) | 461 (0.95%) | <0.001 | 1,102 (2.28%) | 873 (1.81%) | <0.001 |
| 5 years | 862 (1.78%) | 636 (1.32%) | <0.001 | 1,438 (2.97%) | 1,209 (2.5%) | <0.001 |

**Supplementary Table 1. Breakdown of dry eye disease (DED) outcome ascertainment by diagnostic coding and topical therapy initiation.** Values represent patients meeting each component of the composite outcome at 1-, 3-, and 5-year follow-up. Because patients could contribute to both diagnostic coding and topical therapy initiation, categories are not mutually exclusive, and percentages may not sum to the total composite outcome. Total cohort sizes vary as they represent matched individuals to different control groups.
